# Draft Genome Sequence of the Aspergillus terreus High-Itaconic-Acid-Productivity Strain IFO6365

**DOI:** 10.1128/MRA.00080-20

**Published:** 2020-04-16

**Authors:** Hiro Takahashi, Tomoyuki Minami, Mitsuyasu Okabe, Enoch Y. Park, Tsukasa Fujimoto, Anna Takahashi, Masataka Murase, Shuichi Fukuyoshi, Kenji Satou, Shin Kanamasa

**Affiliations:** a Graduate School of Medical Sciences, Kanazawa University, Kanazawa, Ishikawa, Japan; b Graduate School of Horticulture, Chiba University, Matsudo, Chiba, Japan; c Fundamental Innovative Oncology Core Center, National Cancer Center, Tokyo, Japan; d Graduate School of Bioscience and Biotechnology, Chubu University, Kasugai, Aichi, Japan; e Aureo Co., Ltd., Tokyo, Japan; f Research Institute of Green Science and Technology, Shizuoka University, Shizuoka, Japan; g Graduate School of Integrated Science and Technology, Shizuoka University, Shizuoka, Japan; h Graduate School of Science and Technology, Shizuoka University, Shizuoka, Japan; i Faculty of Information Technologies and Control, Belarusian State University of Informatics and Radio Electronics, Minsk, Belarus; j Institute of Medical, Pharmaceutical, and Health Sciences, Kanazawa University, Kanazawa, Ishikawa, Japan; k Faculty of Biological Science and Technology, Institute of Science and Engineering, Kanazawa University, Kanazawa, Ishikawa, Japan; l College of Bioscience and Biotechnology, Chubu University, Kasugai, Aichi, Japan; University of California, Riverside

## Abstract

Itaconic acid is an important organic acid used in the chemical industry. Aspergillus terreus strain IFO6365 is one of the highest-yielding itaconic acid-producing wild-type strains. Here, we report the draft genome sequence of IFO6365, enhancing the understanding of the role and biosynthesis of itaconic acid in this fungus.

## ANNOUNCEMENT

Itaconic acid is an organic acid that is widely used as a raw material in the production of different chemicals. Industrially, the compound is produced by Aspergillus terreus due to its high yield (1). A. terreus IFO6365 is one of the strains with the highest yields of itaconic acid, despite being a wild-type strain (2). We previously cloned a gene encoding *cis*-aconitic acid decarboxylase that was essential for itaconic acid biosynthesis in this fungus (3). The Broad Institute sequenced the A. terreus NIH2624 genome at 11.05× coverage using whole-genome shotgun sequencing (4); the draft genome assembly, consisting of 26 scaffolds, is available in the EnsemblFungi database (https://fungi.ensembl.org/Aspergillus_terreus). We recently sequenced the genome of A. terreus TN-484 (5, 6), an itaconic acid productivity mutant derived from IFO6365. To obtain parental strain information for TN-484, genomic analysis of IFO6365 was performed.

IFO6365 cells, cultured in potato dextrose broth at 30°C, were homogenized using a μT-12 bead crusher (Taitec Co., Saitama, Japan), and genomic DNA was isolated using a NucleoSpin plant II kit (TaKaRa Bio, Inc., Shiga, Japan). Using a NEBNext Ultra II FS DNA library preparation kit for Illumina (New England Biolabs, Ipswich, MA, USA), DNA fragmentation (average fragment length, 600 bp) using DNA fragmentase, end repair, A tailing, adaptor ligation, PCR, and library purification were conducted for library preparation according to the manufacturer’s instructions. Sequencing of the TruSeq DNA library (paired-end 300-bp reads) generated 17,711,412 reads. The adapter sequence removal and low-quality base trimming, followed by *de novo* assembly, were conducted using CLC Genomics Workbench version 12.0.3 (Qiagen, Valencia, CA, USA) with default parameters. Then, reference-guided scaffolding of the draft genome was conducted using RaGOO version 1.1 (7), with default parameters, and the A. terreus NIH2624 genome sequence (GenBank accession number GCA_000149615). Among the RaGOO-generated scaffolds, a concatenated, unlocalized scaffold was discarded, because the scaffold consisted of short, highly fragmented contigs that had no homology to the reference sequence. The resulting genome assembly had a length of 28,633,404 bp and was divided into 23 scaffolds. The contig *N*_50_ value was 2,163,102 bp, the GC content was 53.1%, and the genome coverage was 170.3×. Of the 290 benchmarking universal single-copy ortholog (BUSCO) genes, 98.2% (including 1.0% duplicated genes) were found in the assembly, as calculated by BUSCO version 3.1.0 (8) using the parameter sp aspergillus_terreus. Coding region prediction for the scaffolds was conducted using AUGUSTUS version 3.2.2 (9) with the following parameters: noInFrameStop=true, genemodel=complete, and species=aspergillus_terreus. The estimated number of genes in the draft genome was 10,189. Gene annotation was also performed using OmicsBox version 1.2.4 (Qiagen) (10) with default parameters. This genomic information could provide insights into the genetic basis of itaconic acid production by IFO6365 origin mutants, such as TN-484 (5, 6).

### Data availability.

The draft genome sequence for strain IFO6365 has been deposited in GenBank/ENA/DDBJ under accession number BLJY00000000 (contig accession numbers BLJY01000001 to BLJY01000023). The SRA/DRA/ERA accession number is DRA009452.
